# Distribution of Class I Integron and *smqnr* Resistance Gene Among *Stenotrophomonas maltophilia* Isolated from Clinical Samples in Iran

**Published:** 2017

**Authors:** Mohammadali Malekan, Bahman Tabaraie, Ladan Akhoundtabar, Parviz Afrough, Ava Behrouzi

**Affiliations:** 1. Microbiology Research Center, Pasteur Institute of Iran, Tehran, Iran; 2. Kousha Faravar Giti, Industrial Research Institute of Biotechnology, Tehran, Iran; 3. Department of Microbiology, Azad University of Jahrom, Jahrom, Iran; 4. Department of Mycobacteriology and Pulmonary Research, Pasteur Institute, Tehran, Iran

**Keywords:** Resistance, *Stenotrophomonas maltophilia*, Trimethoprim-sulfamethoxazole

## Abstract

**Background:**

*Stenotrophomonas maltophilia (S. maltophilia)* is a multiple-antibiotic-resistant opportunistic pathogen that is being isolated with increasing frequency from patients with health-care-associated infections. *S. maltophilia* is inherently resistant to most of the available antimicrobial agents. Spread of resistant strains has been attributed, in part, to class I integrons. *In vitro* susceptibility studies have shown trimethoprim-sulfamethoxazole and new floroquinolones as two important agents with activity against these organisms.

**Methods:**

150 isolates of *S. maltophilia* were isolated from clinical samples such as respiratory discharges, sputum, and catheter and hospital environments. These isolates were also subjected to susceptibility testing and polymerase chain reaction for four groups of genes including *int* encoding integron elements, *sulI* and *sulII* encoding trimethoprim-sulfamethoxazole resistance and *smqnr* encoding quinolone resistance.

**Results:**

The rate of resistance to trimethoprim-sulfamethoxazole was up to 27 (18%) and the highest resistance to quinolone family belonged to ofloxacin (20%) and the lowest rate was for gatifloxacin (16%). The results showed that 14% of isolates contained integron elements concomitantly with *sulI* and *sulII* genes.

**Conclusion:**

Resistance rate of *S. maltophilia* to co-trimoxazole and fluoroquinolones and detection of integron elements between isolates in this study showed that this rate corresponded to other data obtained from other studies.

## Introduction

*Stenotrophomonas maltophilia* *(S. maltophilia)* is an aerobic, nonfermentative, gram-negative, catalase-positive and oxidase-negative bacterium. *S. maltophilia* is ubiquitous in aqueous environments including water, urine, or respiratory secretions, soil and plants ^1^. This bacterium causes nosocomial infections in immuno-compromised patients and frequently colonizes breathing tubes such as endotracheal or tracheostomy tubes, the respiratory tract and urinary catheters. Infection easily commences by the presence of prosthetic material (plastic or metal), and the most effective treatment is removal of the prosthetic devices. Therefore, growth of *S. maltophilia* isolated from respiratory or urinary specimens in microbiological media is difficult to interpret and not a proof of infection. However, isolation of *S. maltophilia* from sterile body regions (*e.g*., blood) usually represents true infection. In immunocompetent individuals, *S. maltophilia* is a relatively unusual cause of pneumonia, urinary tract infection, or blood stream infection. *S. maltophilia* is naturally resistant to many antibiotics (including all carbapenems) and often difficult to eradicate. Although resistance has been increasing, many strains of *S. maltophilia* are sensitive to co-trimoxazole and ticarcillin ^2^. It is not usually sensitive to piperacillin, and sensitivity to ceftazidime is variable. *S. maltophilia* is resistant to many β-lactams, β-lactamase inhibitors, and aminoglycosides ^3,4^.

A recent survey has indicated that newer fluoroquinolones demonstrated good efficacy against these bacteria. The most active antimicrobials were levofloxacin and gatifloxacin in which resistance rates were reported to be 6.5 and 14.1%, respectively ^5^. Because of low resistance levels (∼5%), trimethoprim-sulfamethoxazole (TMP/SMX) has remained the choice of antimicrobial therapy against *S. maltophilia* infections worldwide. Although there are a few surveillance studies for Stenotrophomonas infections, resistance to TMP/SMX appears to be emerging, and recent *in vitro* modeling studies have shown that combination therapies by TMP/SMX plus ciprofloxacin or TMP/SMX plus tobramycin exhibit a greater killing capacity than TMP/SMX alone ^2,6^. In addition, *S. maltophilia* can acquire antimicrobial resistance through integrons, transposons, and plasmids. Class 1 integrons have been characterized from *S. maltophilia* strains isolated in Argentina and Taiwan, which indicates that they contribute to TMP/SMX resistance through the *sulI* gene carried as part of the 3′ end of the class 1 integron ^7^. *Sul* genes have been reported to contain class 1 integrons and insertion element common region (ISCR) elements that are responsible for high rate of resistance to TMP-SMX in *S. maltophilia* ^8,9^.

Recent analysis of *S. maltophilia* has identified a novel family of resistance genes (*smqnr*) encoding proteins containing pentapeptide repeats, which confer low-level resistance to quinolones ^10^. Qnr gene exists in several bacterial genera and is located in *S. maltophilia* chromosome, designated as smqnr which encoded a protein that contributes to intrinsic resistance to quinolones ^9^. This gene could be plasmid-borne and results in high resistance to quinolones in wild type and mutant bacteria ^11,12^.

Examination of K279a and another *S. maltophilia* genome sequence (R551-3) also identified a novel family of genes (*smqnr*) that encode proteins with homology to the Qnr quinolone protection proteins found in the Enterobacteriaceae ^5^. These Smqnr proteins (QnrA, B and S) in which pentapeptide repeats could be found, confer low-level quinolone resistance by protecting DNA gyrase and topoisomerases. In Enterobacteriaceae members, Smqnr proteins are usually located in association with other resistance determinants on large plasmids ^13^. The clinical importance of plasmid-mediated quinolone resistance is uncertain, although it is postulated that it may help stabilize or select for mutations in the Quinolone Resistance-Determining Region (QRDR) of DNA gyrase and topoisomerase, which then confers high-level quinolone resistance ^13^. This study could determine the relation between dissemination and increasing antibiotic resistance of *S. maltophilia* isolated from hospital and patient’s samples.

Some studies have been performed in Iran that focused on *S. maltophilia* antibiotic resistance. In one typical study, 3% of 895 isolates were resistant to co-trimoxazole.

## Materials and Methods

### Bacterial strains

During a two year period between 2012 to 2014, 150 isolates of *S. maltophilia* were collected from different clinical settings in Tehran, Iran and clinical samples like respiratory samples, ventilator associated pneumonia, discharges of patients, surgery devices and catheters. Body fluids were inoculated to BACTEC media. BACTEC system was pereferred for better performance in growth and identification of microbial agents because detection procedure was based on on-time and precise computed mechanism.

### Antibiotic susceptibility testing

Susceptibility testing was performed by disc diffusion methods (Mast diagnostics) and E-test (AB Bio-disk) for trimethoprim-sulfamethoxazole, ciprofloxacin, ofloxacin, gatifloxacin and moxifloxacin on Muller Hinton agar as described by Clinical and Laboratory Standards Institute. Resistant strains to trimethoprim-sulfamethoxazole and quinolones were stored at −70*°C*.

### Polymerase chain reaction amplifications

Extraction and purification of DNA from bacterial colonies and plasmids was accomplished by commercial extraction kits from the isolates (QIAmp mini kit from Qiagen). Polymerase Chain Reaction (PCR) was carried out in 50 *ml* containing 2 *μl* template DNA, 5 *μl* 10× concentrated PCR buffer, 1 *μl* of each appropriate primer, 10 *μl* dNTPs, 1 *μl* Taq DNA polymerase, and 21 *μl* sterilized distilled water ^10^. Primer designation and sequences are shown in table 1.

**Table 1.** List of primers used in this study

**Primer**	**Sequence (5′–3′)**	**Amplicon size (*bp*)**	**Reference**
***smqnr*F**	ACACAGAACGGCTGGACTGC	817 *bp*	8
***smqnr* R**	TTCAACGACGTGGAGCTGT		
***sulI* F**	GACGGTGTTCGGCATTCT	420 *bp*	
***sulI* R** ***sul2*F** ***sul2*R**	TTTGAA GGTTCGACAGC GCAGGCGCGTA AGCTGA GGCTCGTGTGTGCGGATG	450 *bp*	2
***int*F**	CGGATGTTGCGATTACTTCG	510 *bp*	7
***int*R**	CGGATGTTGCGATTACTTCG		

## Results

### Antimicrobial susceptibility testing

Strains were tested for susceptibility to trimethoprim-sulfamethoxazole, ciprofloxacin, ofloxacin, gatifloxacin and moxifloxacin. The rate of resistance to TMP/SMX was up to 27 (18%) and this rate for quinolone family was: ciprofloxacin 27 (18%), gatifloxacin 24 (16%), moxifloxacin 25 (17%), and ofloxacin 30 (20%). The TMP/SMX-resistant isolates possessed MICs >32 *μg/ml*, whereas the sensitive controls possessed TMP/SMX MICs ranging from 0.5 to 2 *μg/ml*. MIC for ciprofloxacin resistant strains was >4 *μg/ml* and for gatifloxacin resistant strains, moxifloxacin resistant strains and ofloxacin resistant strains of *S. maltophilia* was >8 *μg/ml* (Table 2).

**Table 2.** Antimicrobial susceptibility testing results

**Antibiotic**	**No. (Percent of total Resistant isolates)**	**No. (Percent of total Susceptible isolates)**	**MIC (*μg/ml*)**
**Trimethoprim-sulfamethoxazole**	27 (18%)	123 (82%)	>32
**Ciprofloxacin**	27 (18%)	123 (82%)	>4
**Gatifloxacin**	24 (16%)	126 (84%)	>8
**Moxifloxacin**	25 (17%)	125 (83%)	>8
**Ofloxacin**	30 (20%)	120 (80%)	>8

### Distribution of class I integron

**Figure 1.**
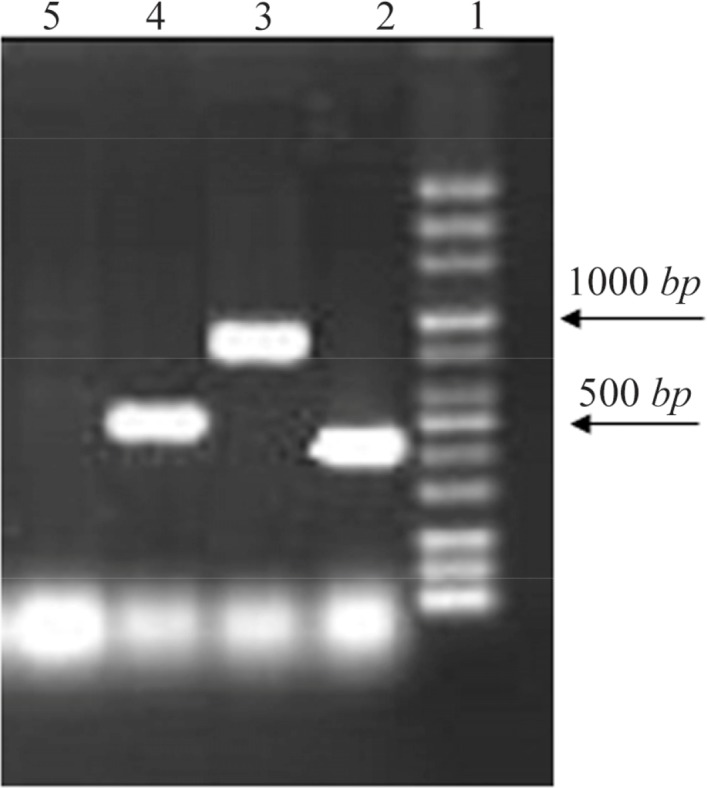
PCR results from right: lane 2, (*sulI*, 420 *bp*); lane 3 (*smqnr* 817 *bp*); lane 4 (*int*, 510 *bp*); lane 5 negative control.

**Figure 2.**
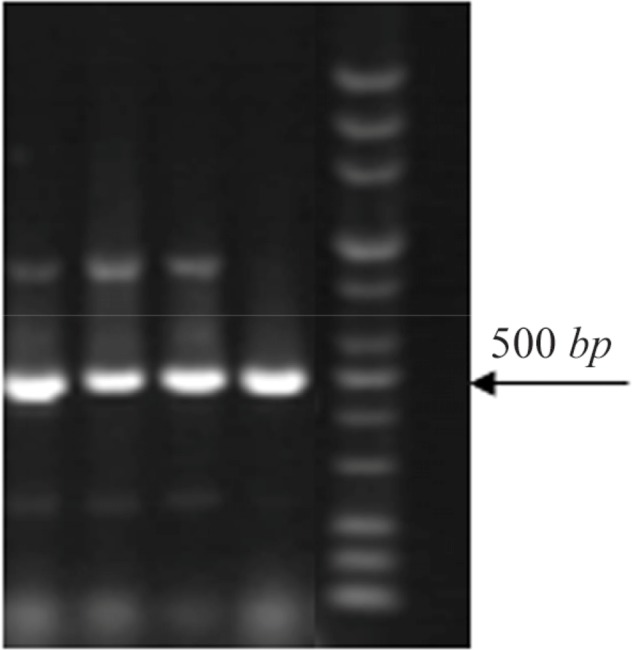
PCR amplification of *sulII* genes (450 *bp*).

14% of strains which were resistant to TMP/SMX contained integron class 1 using primers *int I* F, *int I*R (5′ conserved region) with DNA bands of 510 *bp* (Figure 1). Out of the 27 TMP/SMX-resistant *S. maltophilia* isolates analyzed, 7 isolates possessed the *sulI* gene. *SulI* gene was located as part of the 3′ end of a class 1 integron. None of the trimethoprim-sulfamethoxazole-susceptible *S. maltophilia* isolates yielded positive *sulI* PCR products. Out of 27 TMP/SMX-resistant *S. maltophilia* isolates, 12 isolates carried *sulII* gene using *sulII*F and *sulII*R primers (Figure 2). None of the TMP/SMX-susceptible *S. maltophilia* isolates displayed positive *sulII* PCR products. Of the 27 TMP/SMX-resistant *S. maltophilia* isolates, 5 strains concomitantly contained *sulI* and *sulII* genes. Out of the 106 resistant *S. maltophilia* isolates,16 (10%) isolates contained *smqnr* genes (Figure 1).

## Discussion

In this study, two groups of antibiotics including trimethoprim-sulfamethoxazole and some of quinolone members such as ciprofloxacin, gatifloxacin, moxifloxacin and ofloxacin were selected as well-known antimicrobial agents against *S. maltophilia*. An attempt was made to analyze genetic determinants responsible for drug susceptibility pattern of 150 *S. maltophilia* strains isolated during a 6 month period in 2010 collected from different places of hospital and clinical samples like blood and respiratory samples, ventilator associated pneumonia, discharges of patients, surgery devices and catheters. The results showed that the resistance of these *S. maltophilia* isolates to TMP/SMX and quinolone has slightly risen. By comparison in our study, it can be concluded that 44% of SXT resistant isolates contained large plasmids including *sulI* genes.

Out of these SXT resistant isolates, 27% of them carried *sulI* gene in class 1 integron. These results implied that most of SXT resistant strains contain plasmids for SXT resistance. This plasmid could usually be observed between Enterobacteriaceae members. Most studies of the location and dissemination of *sulII* genes have concentrated on Enterobacteriaceae, such as *Escherichia coli (E. coli)* and *Salmonella enteric* ^8^. The data presented in this study showed that *sulII* gene may spread by Enterobacteriaceae origins among *S. maltophilia* strains. These data suggest that microbiology laboratories need to carefully monitor *S. maltophilia* strains which show resistance to TMP/SMX, because they have the potential to increase by means of mobile genetic elements.

Betrieu *et al* showed that 91% of strains were susceptible to SXT and MIC ^14^. Based on a study in 2001, rates of resistance to SXT ranged from 2% in Canada and Latin America to 10% in Europe ^15^. In another study performed in Saudi Arabia in 2006, two resistant cases to SXT were reported. Both isolates were resistant to TMP-SXT (MIC >8/152 *μg/ml* by MicroScan system and MIC >32 *μg/ml* by E-test strip). The two isolates were also resistant to gentamicin (MIC >8 *μg/ml*), both meropenem and imipenem (MIC >16 *μg/ml*) and ciprofloxacin (MIC >4 *μg/ml*). They were sensitive to ceftazidime (MIC <2 *μg/ml*) and ticarcillin-clavulanate (MIC=16/2 *μg/ml*). The sensitivities to amikacin, chloramphenicol, tetracycline, levofloxacin, aztreonam and piperacillin-tazobactam were variable between the two isolates ^2^. In a study performed in England in 2005, it was indicated that none of *S. maltophilia* isolates from salad was resistant to trimethoprim-sulfamethoxazole, ciprofloxacin, but these isolates concomitantly were resistant to chloramphenicol; 7 (78%) to piperacillin/tazobactam; 5 (56%) to ceftazidime, and 2 (22%) to gentamicin. But it should be considered that the number of samples (salad) in the mentioned study was so small and cannot be compared with this study because our study was about clinical samples and there were many specimens ^16^.

It can be concluded that as the time passes, the rate of resistance of first line effective antibiotics to *S. maltophilia* developes and many isolates should be considered for testing in laboratory. The most significant study ever performed on susceptibility of *S. maltophilia* was a study in 1999 in Department of Microbiology and Immunology, Queen’s University, Kingston, Ontario K7L 3N6, Canada entitled “Multiple Antibiotic Resistance in *Stenotrophomonas maltophilia*: Involvement of a Multidrug Efflux System”.

## Conclusion

In this study, the mechanisms of resistance and percentage of susceptibilities to antibiotics were indicated ^4^. There are some studies performed in Iran which focused on *S. maltophilia* isolates and its antibiotic resistance. In a study in 2011 among a total of 12922 blood specimens, 2300 specimens had a positive blood culture (17.7%); the specimens were collected early at hospitalization and as a result, blood samples were collected before initiation of any treatment. Not considering fungal growth, 21 microorganisms were recognized, with *S. maltophilia* being the most common one (895 specimens; 38.9%). There were 95 sensitive and 5 resistant species in both the disk diffusion method and E-test for co-trimoxazole ^25^.
